# Plasma Processing with Fluorine Chemistry for Modification of Surfaces Wettability

**DOI:** 10.3390/molecules21121711

**Published:** 2016-12-13

**Authors:** Veronica Satulu, Maria Daniela Ionita, Sorin Vizireanu, Bogdana Mitu, Gheorghe Dinescu

**Affiliations:** National Institute for Lasers, Plasma and Radiation Physics, Atomistilor 409, Magurele-Bucharest, Ilfov 077125, Romania; veronica.satulu@infim.ro (V.S.); daniela.ionita@infim.ro (M.D.I.); s_vizi@infim.ro (S.V.); mitub@infim.ro (B.M.)

**Keywords:** plasma processing, fluorinated thin films, nanostructured surfaces, superhydrophobic surfaces

## Abstract

Using plasma in conjunction with fluorinated compounds is widely encountered in material processing. We discuss several plasma techniques for surface fluorination: deposition of fluorocarbon thin films either by magnetron sputtering of polytetrafluoroethylene targets, or by plasma-assisted chemical vapor deposition using tetrafluoroethane as a precursor, and modification of carbon nanowalls by plasma treatment in a sulphur hexafluoride environment. We showed that conformal fluorinated thin films can be obtained and, according to the initial surface properties, superhydrophobic surfaces can be achieved.

## 1. Introduction

Fluorine atoms are the most electronegative atoms and upon combination with carbon can form various compounds with remarkable properties for a broad range of applications, such as the modification of surfaces wettability, waterproof/water repellent textiles, increase durability, and decrease of friction coefficient [1], optical applications [2], changing the electronic states of dielectric multilayer [3], etc.

In particular, wettability is an important property of a surface, wetting or non-wetting behavior having an impact on a wide range of scientific and technological fields. In respect to water, the wettability of a surface is discussed in terms of hydrophilic, hydrophobic, or superhydrophobic character. The typical characterization of wettability requires contact angle measurements. They indicate, quantitatively, the degree of wetting, from analyzing the shape of a liquid drop placed on the surface. Hence, small contact angles (<90°) show a high wettability—hydrophilic surface in case of water, a high contact angle (>90°) corresponds to low wettability, which means hydrophobic surface, while a very large contact angle (>150°, is characteristic for a super-hydrophobic surface [4,5]. The super-hydrophobic surfaces are required in biomedicine to reduce the bacterial adhesion to implants, to reduce clotting and fouling on cardiac stents, in wound healing, in textile industries for advanced multifunctional clothes [6,7], as anti-sticking exterior paints for buildings, in piping and boat hulls, for self-cleaning windows, in microfluidics, and sensors [8,9].

Wettability is a complex phenomenon and depends on the chemical composition and the topography of the surface. The chemical composition dictates the strength of surface interaction with the liquid molecules. As such, a surface containing polar chemical groups interacts strongly with the polar water molecules, thus leading to a hydrophilic character. Oppositely, surfaces that are chemically inert with respect to the liquid promotes a hydrophobic behavior. In this respect, the fluorination procedures play a key role in the fabrication of hydrophobic surfaces. In addition, the surface topography is very important for wetting. In a simple view the capillarity effects enhance/impede on water spreading inside the nano-micro-features of a rough surface of a hydrophilic/hydrophobic material, thus enhancing the respective type of wetting: surfaces of hydrophilic materials become more hydrophilic, and those of hydrophobic materials becomes more hydrophobic when their surface roughness is increased. This is quantitatively described by the Cassie model [10]. Therefore, an efficient strategy to obtain very hydrophobic or superhydrophobic surfaces is to combine fluorination with nanostructured surfaces, for example, to coat surfaces with nano-micro-topography with very thin fluorinated layers.

Nevertheless, the coating of surfaces with thin films of fluorinated compounds is challenging. The methods for fluorocarbon film deposition are based on formation of low weight polymer fragments that travel in vacuum environment from a precursor source to a substrate, forming at the surface a polymer network. The precursors are created from fluoro-containing solid materials (Polytetrafluoroethylene (PTFE), Parylene-F) by thermal or sputtering effects, or from fluorocarbon gases (C_4_F_8_, CF_4_, NF_3_, SF_6_) by gas-phase dissociation processes.

The main methods using solid targets of fluorine-containing materials are electron beam irradiation [11], thermal chemical vapor deposition [12], and vacuum evaporation [2]. These methods can only be used in special cases where polymers with a low molecular weight evaporate as polymer units and condense on the substrate without a reaction. The evaporation temperature conducts to fragmentation of most of polymers and the gaseous resulted fragments condense on the substrate without creating a proper polymer network [13]. Another method using solid targets is laser ablation [14]. The main drawbacks of the laser ablation technique are the limited deposited area and the lack of uniformity control. Alternatively, advantages like better uniformity of the thin films and larger deposition areas are obtained by radiofrequency (RF) magnetron sputtering [15,16,17,18,19], a plasma method in which volatile fragments sputtered from a polymeric target are released into the plasma volume and serve as gaseous precursors for the polymerization process at the surface [13].

In respect to the methods using fluorocarbon gases as precursors, the plasma-enhanced chemical vapor deposition technique (alternatively named as plasma polymerization) is by far the most used. A typical example is the formation of thin fluorocarbon films from tetrafluoroethane (freon-C_2_F_2_H_4_). Tetrafluoroethane, a well-known refrigerant under the trade name R134a, is one of the most used fluorinated gases in plasma discharges that can easily lead to thin film deposition [20,21,22] and polymers in porous absorbent industry. The obtained polymers have high molecular weight, low densities, and despite weak mechanical performance and poor adhesion to the substrate, they have special characteristics as thermal and electrical insulators. As another example, SF_6_ plasmas can be used in processing for adding new functionalities at surface. SF_6_ plasmas are sources of atoms, ions and fluorinated radicals and were used for functionalizing carbon nanostructures [23] in particular for carbon nanotubes [24].

The present work illustrates the strategy for obtaining highly hydrophobic, or even super hydrophobic coatings by using fluorine containing discharges. Namely, magnetron sputtering of polytetrafluoroethylene, plasma assisted chemical vapor deposition of tetrafluoroethane and sulphur hexafluoride plasma treatment were employed for deposition on, or treatment of, surfaces. For a proper chemical, morphological, and topographical characterisation smooth silicon samples were used first. Secondly, we applied the same fluorination procedures to highly nanostructured surfaces, specifically to carbon nanowalls (CNW). The properties of the smooth and highly nanostructured fluorinated surfaces, with focus on wettability, are comparatively discussed according to the methods used to add fluorine to the surface.

## 2. Results

### 2.1. Fluorinated Thin Films Obtained by Magnetron Sputtering of PTFE Targets

#### 2.1.1. Chemical Composition of the Sputter-Deposited Thin Films

• X-ray Photoelectron Spectroscopy Spectra

X-ray Photoelectron Spectroscopy (XPS) survey spectra revealed carbon and fluorine as the main elements both for the PTFE target and PTFE-like material, while oxygen and nitrogen are present as contaminants in the case of plasma deposited material. The atomic composition of conventional PTFE and PTFE magnetron sputtered material at 50 W, as determined from the XPS survey spectra interpretation, is reported in Table 1. The ratio between fluorine and the carbon atomic concentrations (F/C ratio) are also included.

For the conventional PTFE, we found upon XPS measurements that F/C ratio is 2.16, while for RF magnetron deposition of PTFE-like material the F/C ratio is 1.08 [25,26]. This lower value of F/C ratio for the plasma deposited PTFE could be explained by two reasons. First, one may refer to the magnetron sputtering deposition process were the fluorine atoms ejected from the target can be pumped out from the deposition chamber, either in atomic form, or upon recombination with a volatile species. Secondly, the carbon contamination which is unavoidable in the natural environment [27] contributes to a higher amount of detected carbon in the XPS spectra at the surface, especially for those materials which are plasma deposited due to the unsaturated chemical bonds on the surface.

**Figure 1:**
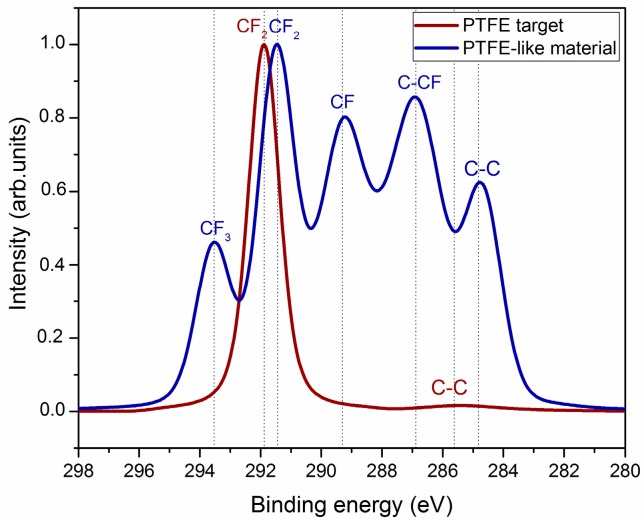
High-resolution spectra of C1s region for PTFE target (**red line**) and magnetron-sputtered PTFE material (**blue line**).

The high-resolution C1s spectra recorded for the initial PTFE target and that of magnetron-sputtered PTFE material are displayed in Figure 1. The deconvolution of C1s spectrum of conventional PTFE (red line) reveals two components, corresponding to CF_2_ bond at 292 eV and C–C bond at 285 eV, suggesting very simple chemical structure of the conventional PTFE [25,28].

Regarding the material deposited from the sputtered PTFE target, the C1s spectrum is decomposed in five components (C1 to C5), assigned as follows: CF_3_, CF_2_, and CF functional groups at 293 eV, 291 eV, and 289 eV, respectively, as well as C–CF and C–C chemical bonds at 287 eV and 285 eV [9,12,23]. Such decomposition reveals the complex structure of the magnetron-sputtered PTFE materials in respect to the conventional ones: beside chain fragments as CF_2_, branching fragments as CF and chain ending fragments as CF_3_ are present. It points towards a pronounced cross-linking in the deposited PTFE layers. This is explained by the plasma process: the target bombardment with Ar^+^ ions, causes sputtering of various fragments from the PTFE target, their ejection into the plasma volume, followed by the transport to substrate and growth of a cross-linked and defect rich film.

• Fourier Transform Infrared Spectroscopy Fingerprint

**Figure 2:**
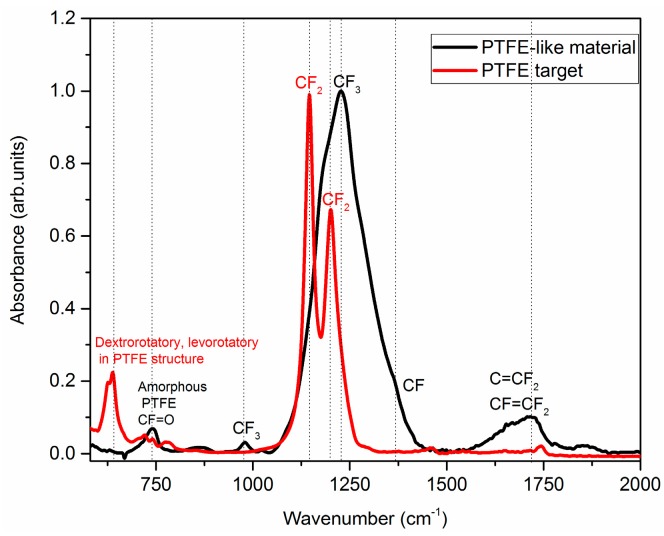
Fourier Transform Infrared (FTIR) spectra of the PTFE target (**red curve**) and PTFE-like material obtained by radiofrequency (RF) magnetron sputtering (**black curve**).

The Fourier Transform Infrared (FTIR) Spectroscopy technique allowed the investigation of the functional groups present in the material. In Figure 2 the FTIR spectra of the PTFE target, as well as of the magnetron-sputtered PTFE-like material are presented. The infrared (IR) spectrum of PTFE target indicate the most intense bands at 1145 cm^−1^ and 1201 cm^−1^ which are assigned to CF_2_ symmetric and asymmetric stretching bonds [29]. The well-known helical structure of the PTFE material is proved by the presence of two bands at 626 cm^−1^ and 638 cm^−1^, associated to laevorotatory and dextrorotatory chirality of the PTFE structure, which denotes the partial crystalline structure of the initial target [25]. The IR spectrum of the PTFE-like material displays several additional IR peaks compared to that of the conventional PTFE, confirming that its structure is more complex. In more detail, the most intense absorption bands of the as deposited PTFE are broader than the most intense peak at 1227 cm^−1^ and a small shoulder at 1182 cm^−1^; in addition to the CF_2_ contribution, typical for the conventional PTFE, the convoluted band also contains a contribution from CF and CF_3_ functional groups [30], which are peculiar for plasma-deposited materials [13]. Additional peaks observed for the magnetron sputtered PTFE-like materials are assigned as follows: CF_3_ vibrations at 978 cm^−1^, CF_2_ combined asymmetric stretching and rocking deformations at 1464 cm^−1^, CF=O vibrations at 739 cm^−1^ that suggest incorporation of contaminants in the film [25]; and C=CF_2_ or CF=CF_2_ vibrations at 1715 cm^−1^ [29], which point out toward the amorphous structure as well as the cross-linked architecture of the sputtered PTFE layers [2].

#### 2.1.2. Morphological and Topographical Characteristics

• Films Deposited on Flat Surfaces

**Figure 3:**
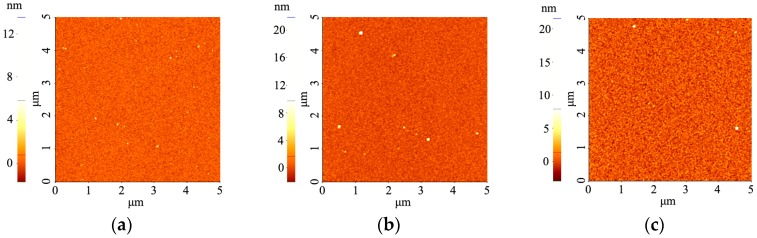
Atomic Force Microscopy (AFM) images of magnetron sputtered PTFE-like thin films deposited for 30 min at various RF powers: (**a**) 50 W; (**b**) 80 W; and (**c**) 110 W.

Atomic Force Microscopy (AFM) images on the PTFE-like materials obtained for various RF power levels are presented in Figure 3. We can easily observe that PTFE thin films present very smooth surface. A slight tendency for surface texturing is observed by increasing the RF power, leading to an almost linear increase of the RMS from 0.50 nm at 50 W to 0.90 nm at 110 W, but still remaining below 1 nm for all investigated conditions.

**Figure 4:**
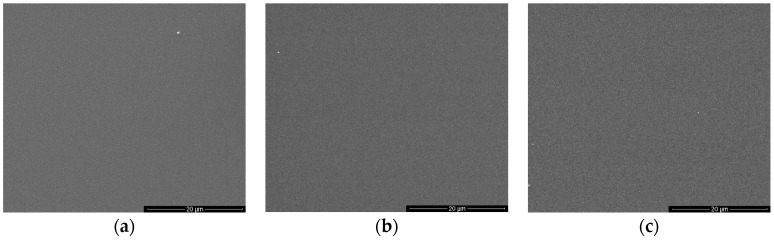
Top view Scanning Electron Microscopy (SEM) images of the magnetron sputtered PTFE materials upon injected RF power to the plasma source: (**a**) 50 W; (**b**) 80 W; and (**c**) 110 W.

Scanning Electron Microscopy (SEM) images of the PTFE-like materials obtained for various powers are shown in Figure 4, revealing again the smoothness of the film’s surface regardless the injected RF power. Even at the highest applied RF power, the material presents a very smooth surface without any morphological features on its surface.

• PTFE Films Deposited on Nanostructured Surfaces (Carbon Nanowalls)

**Figure 5:**
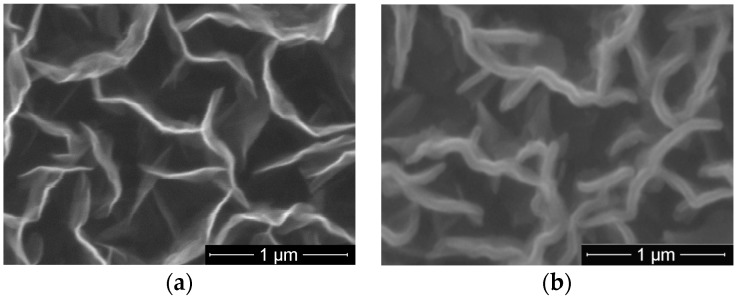
SEM images of carbon nanowalls (CNW) (**a**) as-deposited; and (**b**) covered by PTFE layer deposited by magnetron sputtering.

The second set of fluorinated samples was performed on CNW. A typical SEM image of as-deposited CNW synthesized in Ar/H_2_/C_2_H_2_ plasma at flow ratios of 1400/25/1 sccm is presented in Figure 5a). It shows the specific morphology of this material, with very thin walls (tens of nm thickness) oriented perpendicular to the substrate and having wall lengths up to 1 μm, forming a highly nanostructured porous architecture. After PTFE magnetron sputtering deposition at 60 W for 15 min, one can notice in SEM image shown in Figure 5b that the porous architecture specific for CNW was preserved, but an increase of walls edges up to 100 nm is obvious. It counts for CNW covering by thin layers of PTFE-like film.

### 2.2. Fluorination of Nanostructured Thin Films Obtained by Plasma Assisted Chemical Vapor Deposition or Treatment by Using SF_6_ and C_2_H_2_F_4_ Gases

#### 2.2.1. Chemical Composition of the CNW Materials Exposed to Fluorinated Plasmas

• XPS Spectra

**Figure 6:**
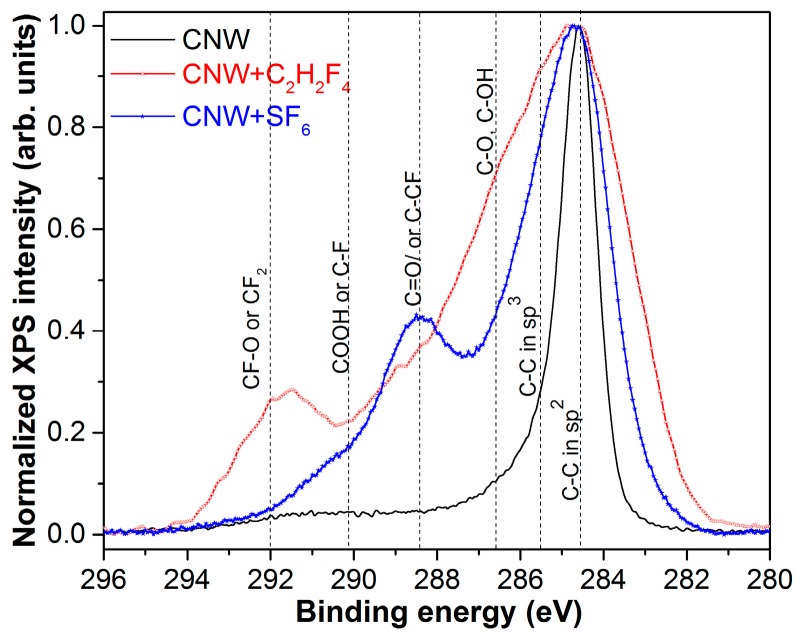
Normalized high-resolution spectra of CNW and treated CNW by SF_6_ and C_2_H_2_F_4_ plasma.

The high-resolution XPS spectra of carbon C1s region for CNW material exposed to fluorinated plasmas (generated in C_2_H_2_F_4_ and SF_6_ gases at 25 sccm) are presented in Figure 6, in comparison to the initial CNW material; they prove the incorporation of various fluorine (CF_x_) groups at the material’s surface.

The XPS spectra could be fitted with seven components (C1–C7) [31], corresponding to the following identification of the bond types: C–Si (283.4 eV); C=C (sp^2^ at about 284.5 eV); C–C (sp^3^) or defects (at 285.5 eV); C–O/C–OH (at approximately 286.6 eV); C=O/ or C–CF (at 288.4 eV), COOH or C–F (la 290 eV) and CF_2_/–CF–O (~292 eV) [32,33]. The concentrations of each carbonic component are presented below in Table 2.

The presence of fluorocarbon chemical groups bonded to the carbon atoms from CNW surface was also reported in [26] upon FTIR investigations of the samples.

**Figure 7:**
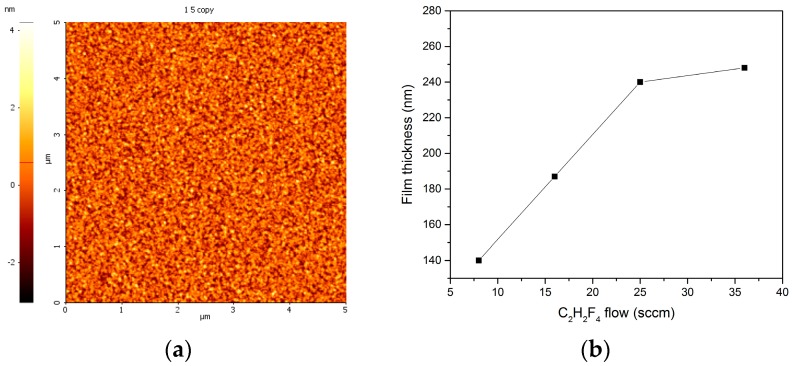
(**a**) Typical topography of fluorocarbon film deposited on flat Si substrate by plasma assisted chemical vapor deposition (PACVD) of C_2_H_2_F_4_ (25 sccm flow); and (**b**) dependence of deposition rate on C_2_H_2_F_4_ flow rates.

It is worth mentioning, with respect to Figure 7, that the formation of CF_2_ bonds is indicated, suggesting new material deposition onto surface.

#### 2.2.2. Morphology and Topography of the Surfaces

• Scanning Electron Microscopy and Atomic Force Microscopy Investigation of C_2_H_2_F_4_ Plasma Treated CNW

In the case of fluorinated plasma treatment, the amount of gases and composition of various species formed in the discharge are very important, determining the dominant processes, among which the most important ones are etching and deposition of a fluorinated film. In our case the treatment was done using various gas flows (2–36 sccm) of the C_2_H_2_F_4_ precursor with a deposition time set to 10 min. The surface of the fluorocarbon film on flat Si substrate, presented in Figure 7a, shows a granular topography. The deposition rates are presented in Figure 7b. They evidence an almost linear increase of the rate at low flows and a saturation tendency at high flows of C_2_H_2_F_4_ precursor.

**Figure 8:**
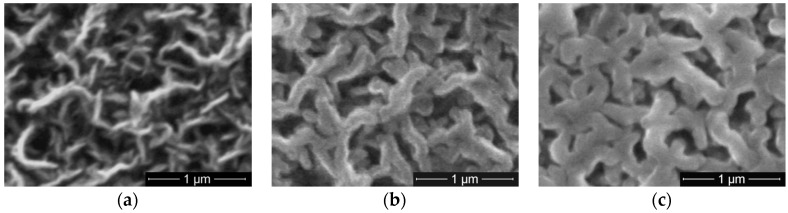
SEM images of CNW treated with C_2_H_2_F_4_ plasma for (**a**) 2 sccm; (**b**) 8 sccm; and (**c**) 36 sccm.

The morphologies of CNW exposed to tetrafluoroethane plasma are presented in Figure 8 for various C_2_H_2_F_4_ flows, and 10 min plasma processing. They evidence that CNW are getting covered with fluorocarbon thin films which conduct to the increase of the walls dimension from tens to hundreds of nm. As well, the walls edges are getting rounded and upon increasing the flow of C_2_H_2_F_4_ we observe that the deposited film tend to cover the space between walls.

**Figure 9:**
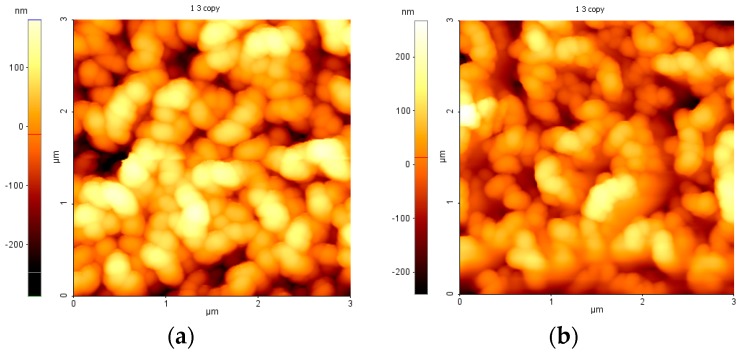
The topography of CNW after fluorocarbon plasma exposure with (**a**) 8 sccm (Root Mean Square (RMS) 69.7 nm); and (**b**) 16 sccm (RMS 80.2 nm) of C_2_H_2_F_4_.

The topographies of fluorocarbon films deposited on top of CNW after 10 min RF plasma treatment in 8 and 16 sccm of C_2_H_2_F_4_ are presented in the Figure 9. Comparing their topography and the average roughness with those of the initial CNW layers (Root Mean Square (RMS) 100.6 nm, not shown here), we can see the rounding of the CNW edge and decreasing the RMS roughness to (70–80 nm) upon Ar/C_2_H_2_F_4_ plasma exposure.

• SEM and AFM investigation of SF_6_ plasma treated CNW

**Figure 10:**
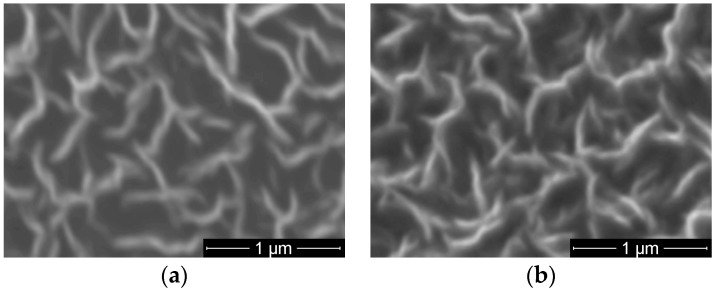
SEM images of CNW treated with SF_6_ plasma for (**a**) 8 sccm; and (**b**) 36 sccm.

The morphologies of 10 min SF_6_ plasma treated CNW are presented in Figure 10. The most significant modification observed upon SF_6_ plasma exposure is the quality of the SEM images, which becomes more blurred, most probably because of surface charging effects due to attachment of fluorine-related radicals onto the CNW.

**Figure 11:**
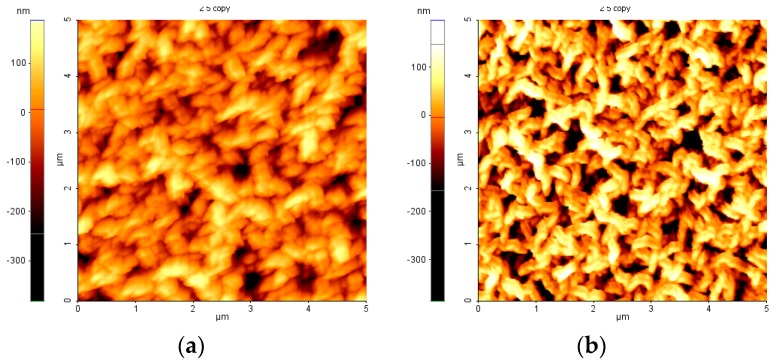
The topography of CNW after SF_6_ plasma treatment using (**a**) 2 sccm; and (**b**) 36 sccm.

While the top view SEM images of CNW treated by SF_6_ plasma evidence the porous architecture similar to that of the initial CNW, the AFM investigation evidence a flattening of the CNW edges, as seen in Figure 11, which suggest a possible etching effect of the CNW material upon 10 min SF_6_ plasma exposure. The roughness RMS of SF_6_ treated CNW decreases from around 75 nm to 43 nm by increasing the SF_6_ flow from 2 to 36 sccm.

**Figure 12:**
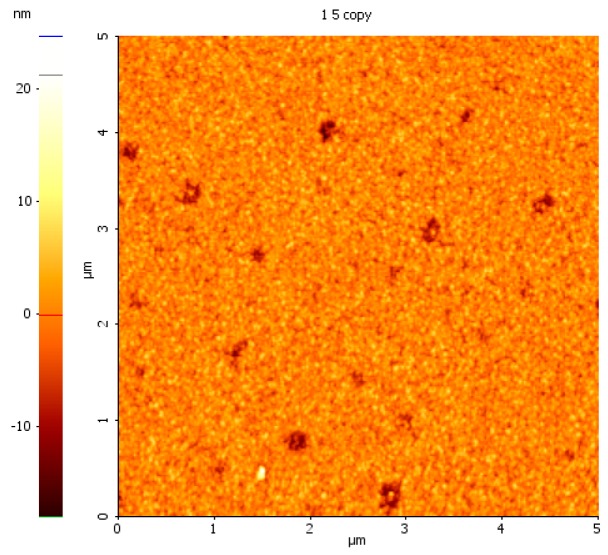
Surface topography of Si after 36 sccm SF_6_ plasma treatment.

The effect of SF_6_ plasma on the Si substrate is the known one, namely a corrosion of the surface which is evidenced in Figure 12 for the case of 10 min plasma generated in presence of 36 sccm SF_6_. In this case, the entire surface become rougher and several holes of more than 10 nm depth are present over the investigated area.

### 2.3. Impact of Surface Modification by Fluorine Containing Radicals on the Wettability

**Figure 13:**
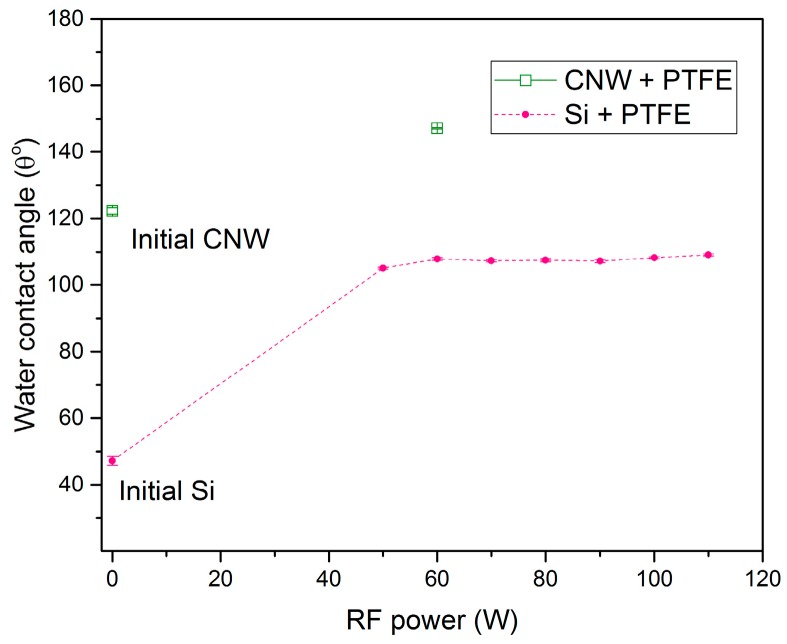
The dependence of the water contact angle on applied RF power for the PTFE-like magnetron-sputtered thin films.

Wettability investigation of PTFE-like materials deposited by magnetron sputtering technique is illustrated in Figure 13. This graph presents the surface contact angle variation against the injected RF power of the PTFE layers deposited on flat surface, namely silicon wafer. These investigations reveal the drastic increase of the water contact angle for the PTFE-like material as compared to the silicon substrate on which the magnetron sputtered PTFE take place. The water contact angles remain almost constant around 110°, the wettability trend revealing only small variations upon increasing RF power.

On the other hand, the PTFE thin film deposited on CNW increase the hydrophobicity of CNW layers from about 122.3° ± 1° of the initial CNW surface to 147.2° ± 0.2°. Such composite PTFE/CNW can be used in various applications to increase the mechanical properties of CNW layers, as electrical insulator/spacers for supercapacitors [34] and are promising for applications related to highly hydrophobic surfaces.

**Figure 14:**
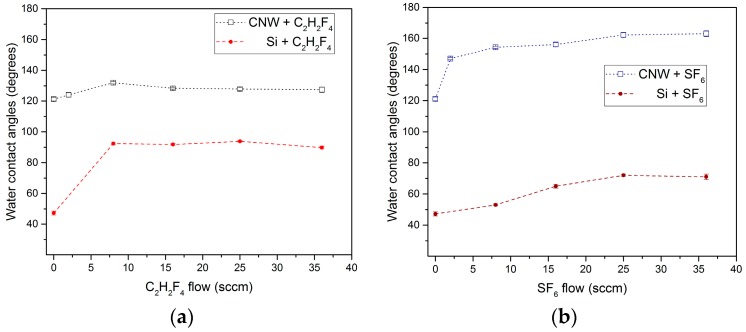
Contact angles of CNW layers and Si wafer after plasma exposure to (**a**) C_2_H_2_F_4_ PACVD process; and (**b**) SF_6_ plasma treatment.

The variation of wettability for the flat smooth surfaces (Si wafer) and highly nanostructured surfaces upon exposure to C_2_H_2_F_4_ and SF_6_ plasmas is presented in Figure 14, as a function of precursor flow introduced into the discharge. As a general observation, the values of the water contact angles are systematically higher upon exposure to fluorinated plasmas. Nevertheless, clear differences are encountered among the two situations (flat vs. nanostructured surfaces), which point out the significance of surface topography in respect to the final wettability characteristics of the surfaces.

For both types of plasmas generated in gaseous precursors, the water contact angles are systematically higher for the nanostructured surfaces, going toward superhydrophobic behavior. For the fluorocarbon deposition, the water contact angle (WCA) on Si is around 90°, while on CNW surface is increasing to around 130°. In the case of SF_6_ plasma treatment, a slight increase of the WCA is encountered for the Si up to 70° for high SF_6_ flows, while WCA exceeding 150° were measured for the CNW treated surfaces.

## 3. Discussion

Fluorinated plasma can be regarded as a reactive medium in which plasma species (like fluorine-containing radicals, negative and positive ions, electrons, etc.) may trigger the processes of polymerization with the formation of clusters and macromolecules or modification of the chemistry of material surfaces. The concentration of fluorinated radicals in the discharge is very important and it depends on the choice of plasma type (magnetron or discharge in gaseous precursors) and conditions (power, pressure, gas flows, etc.). Additionally, the exact mechanisms of incorporation of fluorine in materials is only partially known, and modelling efforts have been devoted to particular systems, like fluorine and graphene networks [35,36]. A particularity of fluorocarbon plasmas is that various particular groups may conduct to the formation of fluoropolymers films while the fluorine atoms and ions are responsible for the etching and corrosion phenomena. The prevalence of one of the above-mentioned processes, namely corrosion or deposition of a fluorinated film, depends greatly on the precursor type and the plasma processes which are mainly governing the C/F ratio in the plasma volume.

In the case of magnetron sputtering the species production is highly depending on the applied RF power, which controls the amount of Ar^+^ ions bombarding the target, as well as their energy influencing the sputtering yield. Additional fragmentation and ionization is produced by the electrons from the magnetron discharge. The chemical composition of the plasma during the RF magnetron sputtering of PTFE target was investigated by means of mass spectrometry and it was observed that most responsible radicals for fluorocarbon thin film growth are CF, CF_2_, and CF_3_ [13]. The positive ions like C_x_F_y_ (x ≤ 4, y ≤ 5) also play an important role in the sputtering process and film deposition. The surface chemistry of the investigated materials in our study, revealed by XPS technique, is dominated by CF_x_ groups and indicates the existing relation with the plasma composition during the magnetron sputtering process. The presence of fluorine containing groups in the film combined with the nano-micro-topography led to a highly hydrophobic character of the CNW coated with PTFE-like material.

In the case of processes based on gaseous precursors (e.g., generated with CF_4_, C_3_F_6_, C_4_F_8_, NF_3_, and SF_6_) the dissociation of molecules is produced mainly by electron collisions. The chemical composition of the plasma during fluorocarbon plasma assisted chemical vapor deposition (PACVD) process is dominated by various species: F, H, C_2_, C_3_, CH, and CF_2_ were detected by optical emission spectroscopy in electron cyclotron resonance and pulsed plasmas [37]. Positive ions CF^+^, C^+^, CF_2_^+^, and CF_3_^+^ were reported by mass spectrometry as being abundant in the discharges of high power impulse magnetron sputtering of a carbon target in CF_4_ and C_4_F_8_ [38]. Investigation of C_3_F_6_ fed plasmas by mass spectrometry-gas chromatography showed in the plasma exhaust by-products containing CF_3_ groups, this radical being detected also in the film by XPS. It was also concluded that the main role in the deposition process is played by gas-surface heterogeneous reactions involving low molecular weight fragments [39]. Our study is in good agreement with these data: the polythetrafluorethane-like thin films synthetized by the PECVD technique presents a complex chemical structure at the surface, including the presence of C–C, C–F, C–O, C–CF bonds.

For the PACVD process, one of the main parameters influencing the deposition is the ratio between the applied power, pressure, and the effective precursor flow [40], which is equivalent to the energy available for volume reactions for each molecule introduced into the plasma. Upon increasing the mass flow rate of the precursor, the deposition rate increased, but the energy of species arriving at the substrate is decreasing, the obtained material has higher roughness and is less conformal to the substrate. When thin layers of fluorocarbon are covering the CNW, the contact angle increases up to 132°, while a slight decrease of the WCA down to 127° is noticed when thick fluorocarbon layers are covering the CNW, conducting to an increased contact area of the water with the surface and decreased volume of the air-pockets.

In the case of sulphur hexafluoride plasma treatment, the WCA on Si is slightly increasing due to a combination of fluorine addition to the surface and surface etching, while significant increase of WCA is encountered for CNW treated surface. Two competing processes are responsible for surface modification of CNW, in a similar manner to those encountered in SF_6_ plasmas used in semiconductor industry [41]. First, the fluorine atoms generated upon SF_6_ dissociation in the plasma conduct to the etching of carbon from CNW edges, leading to their levelling; second, the carbon species released from CNW upon etching recombine in the vicinity of the surface with fluorine atoms and lead to condensing fluorocarbon species which are redeposited on the edge of CNW. Since the amount of material to be redeposited is low, the overall aspect of the CNW remains the same (morphology—SEM), while the chemistry is changed toward a fluorinated carbon one (XPS) with superhydrophobic behaviour.

The data presented in this study evidence several common characteristics of the materials exposed to fluorine containing species, namely the formation of specific CF_x_ bonds originating from the material deposition, in case of magnetron sputtering of PTFE and PACVD of C_2_H_2_F_4_ and, respectively, the attachment of fluorine to the CNW in case of plasma treatment in SF_6_ atmosphere. At the same time, we have shown that the surface topography is a key element in obtaining superhydrophobic surfaces. While the use of plasmas (magnetron sputtering or PACVD techniques) for deposition of fluorocarbon material onto flat substrates conducted to surfaces presenting a hydrophobic character, WCA > 90°, the utilization of nanostructured surfaces as support for the fluorocarbon deposition or sulphur hexafluoride plasma treatment conducts to superhydrophobic behaviour. This is explained by the Cassie-Baxter wetting regime [10], which implies that the liquid is suspended on top of the nanostructured surface, forming air-pockets in the volume between the walls of CNW and drastically reducing the effective contact area [42]. Although the contact angle measurements were performed only in static mode, we could have easily identified the wetting regime due to the slippery behaviour of the surface, which showed that the water droplet is jumping on the superhydrophobic surface upon release. A relevant supplementary video material is presented in support of this aspect.

## 4. Materials and Methods

### 4.1. Substrates

The substrates used in the experiments were smooth silicon wafers (100) or silicon wafers coated with CNW. The CNW were obtained by a low-pressure RF plasma jet from an Ar/H_2_/C_2_H_2_ mixture. The growth parameters for the CNW samples were: gas flows of Ar/H_2_/C_2_H_2_ at a ratio of 1400/25/1 sccm, a working pressure of ~130 Pa, and applied RF power of 300 W. The substrate growth temperature was set at 700 °C. Detailed results regarding the morphology, structure, topography and the surface chemistry of as-obtained CNW can be found in [43,44,45].

### 4.2. Methods

#### 4.2.1. Deposition of PTFE-Like Layers by Magnetron Sputtering

Deposition of fluorinated layers starting from a solid PTFE target was conducted in a spherical stainless steel vacuum chamber evacuated by a turbomolecular/rotary pumping system. For this purpose, the chamber is equipped with a magnetron head (Kurt J. Lesker) mounted at 45° and 9 cm distance in respect to a rotating substrate holder, which serves also as a grounded electrode. The pressure in the chamber was monitored by a Pfeiffer baratron vacuum gauge and the gas flow rates were controlled by electronic mass flow controllers (Bronkhorst Instruments). Silicon wafers and CNW covered Si were used as substrates. The RF sputtering process was conducted in argon (100 sccm) with power values in the range of 50–110 W, and at a working pressure of 6.8 × 10^−1^ Pa. In order to evaluate the growth rate of PTFE layers, the deposition time was varied from 10 min to 35 min, and the layer thickness was measured by AFM. The deposition rate varies almost linearly with the power from 1 nm/min at 50 W to 6.6 nm/min at 110 W.

#### 4.2.2. Surface Fluorination by Plasma Assisted Chemical Vapor Deposition of C_2_H_2_F_4_ and Plasma Treatment in SF_6_ Discharge

The experiments for plasma deposition or treatment onto CNW material were performed in a Bell-Jar reactor [30] separately for CNW synthesis setup [43]. The capacitively-coupled discharge (RF, 13.56 MHz) was generated between two parallel planar electrodes laid 4 cm apart. The upper electrode is connected to the RF power supply, while the grounded electrode is used as substrate holder. Gas mixtures of Ar/(SF_6_ or C_2_H_2_F_4_) were admitted into the chamber at constant Ar flow of 10 sccm and fluorinated gases in a range of 2–36 sccm, leading to a working pressure of 5–20 Pa. The treatment time was 10 min at 20 W applied RF power.

### 4.3. Material Characterization Methods

#### 4.3.1. Chemical Composition of Plasma Fluorinated Samples

Investigations regarding the chemical composition and bonding of the materials obtained in various plasma conditions were conducted by means of X-ray photoelectron spectroscopy (XPS) and Fourier transformed infrared spectroscopy (FTIR). XPS analyses were performed on a K-Alpha Thermo Scientific (Waltham, MA, USA) spectrometer equipped with a hemispherical analyser. For the excitation of photoelectrons, X-ray radiation of an aluminium anode (AlKα, 1486.6 eV) generated at a tube voltage of 12 kV and current emission of 3 mA was used. Peak position was calibrated according to the standard C1s peak (284.6 eV). Survey spectra were recorded to determine the elemental composition of the porous composite membrane surface, while high resolution spectra for C1s, F1s, O1s, and N1s were measured in order to evaluate the chemical bonding of the material.

Chemical bonds of the deposited PTFE-like material were investigated using a JASCO 6300 FTIR spectrometer (Easton, MD, USA). The spectra were recorded in transmission mode in the range 400–4000 cm^−1^, with a resolution of 4 cm^−1^ and an average number of 128 scanning.

#### 4.3.2. Surface Topography and Morphology

The modifications encountered upon exposure of the substrates to various plasma configurations and experimental conditions were evaluated by means of AFM and, respectively, SEM techniques for characterization of surface topography and morphology. AFM images were recorded with a Park Systems XE-100 microscope (Suwon, Korea) operating in non-contact mode, for various areas; herein we presented data recorded over areas of 5 × 5 μm^2^. The analysis of the AFM data was carried out using the “XEI” software from Park Systems (version 1.7.6). First order line by line flattening was applied. Points with larger height (corresponding to the grains) were excluded from this line fitting using a height threshold mask. RMS values were calculated for one image, for each sample. SEM measurements were performed on a FEI S Inspect high-resolution microscope (Hillsboro, OR, USA), operating at an accelerating voltage of 5 kV. In order to prevent charge accumulation on the surface, the samples were coated with a very thin gold layer (5 nm) deposited by the magnetron sputtering technique.

#### 4.3.3. Surface Wettability

The wettability was investigated by water contact angle measurements. The measurements were performed using a CAM 101 optical system (KSV Instrument Ltd., Helsinki, Finland), by the sessile drop method. The experiments were realized at room temperature (25 °C) by placing a drop of about 1 µL of distilled water onto the surface. The behavior of the drop, after its placement on the surface, was studied by capturing the temporal series of drop images (frames) with the charge coupled device (CCD) camera of the instrument. The images selected for calculations consisted of a set of frames, between 10 and 20, recorded in the normal mode, after the drop stabilization on the surface (which takes about 1 s). The Young/Laplace equation was used in the fitting procedure for each image. A value of the contact angle was supplied by the instrument software upon averaging of such a set, and we assigned it to the local point of the surface, where the measurement was performed. Further statistical processing considered the local contact angles obtained from five different places on the surface. The final contact angle, characteristic to the surface, was obtained by averaging the local contact angles, and the error was estimated by the standard deviation of the local values from the average.

## Figures and Tables

**Table 1:** Atomic composition of the investigated materials and F/C ratio of the conventional PTFE and sputtered PTFE-like films.

Sample	F (%)	C (%)	O (%)	N (%)	F/C Ratio
PTFE target	68.40	31.60	-	-	2.16
PTFE-like films	49.70	45.86	3.43	1.01	1.08

PTFE: Polytetrafluoroethylene.

**Table 2:** Positions and concentration of C1s components for CNW exposed to C_2_H_2_F_4_ and SF_6_ plasma, respectively.

Components	Position	Concentration CNW + SF_6_	Concentration CNW + C_2_H_2_F_4_
	(eV)	%	%
C1	283.4	5.6	9.8
C2	284.5	30.7	25.9
C3	285.5	19.5	18.1
C4	286.6	14.4	15.6
C5	288.4	19.1	15.0
C6	290.0	8.6	3.2
C7	292.0	2.1	12.4

## Supplementary Materials

The following are available online at: http://www.mdpi.com/1420-3049/21/12/1711/s1, Video S1: Behaviour of water droplet upon contact with surface of carbon nanowalls treated in sulphur hexafluoride plasma.
